# Cardiovascular surrogate markers and cardiometabolic therapeutics: a viewpoint learned from clinical trials on dipeptidyl peptidase-4 inhibitors

**DOI:** 10.1186/s12933-021-01234-5

**Published:** 2021-02-11

**Authors:** Atsushi Tanaka, Koichi Node

**Affiliations:** Department of Cardiovascular Medicine, Saga University, 5-1-1 Nabeshima, Saga, 849-8501 Japan

**Keywords:** Surrogate marker, Vascular function, Vascular failure, Dipeptidyl peptidase-4 inhibitor

## Abstract

Clinical trials are often performed to investigate the effects of various types of cardiometabolic therapies on cardiovascular surrogate markers, including vascular function and biomarkers. This study platform has the potential to provide information on the suspected actions of drugs and mechanistic insights into their prognostic impact. However, despite using the same class of drugs and similar study designs we are often faced with inconsistent and even conflicting results, possibly leading to some confusion in the clinical setting. When interpreting these results, it is important to investigate what caused the differences and carefully assess the information, taking into account the research situation and the patient population investigated. Using this approach, assessment of the impact on cardiovascular surrogate markers observed in clinical studies from multiple perspectives should help to better understand the potential cardiovascular effects. In this commentary we discuss how we should interpret the effects of cardiometabolic therapeutics on vascular surrogate markers, based on viewpoints learned from the results of clinical trials on dipeptidyl peptidase-4 inhibitors. This learning strategy could also be helpful for appropriate selection of drugs for evidence-based, patient-centric, tailored medication.

## Introduction

Obesity and metabolic disturbances, such as insulin resistance and hyperinsulinemia, are fundamentally associated with the development of vascular failure and atherosclerosis [1]. The metabolic syndrome is therefore a major cause of atherosclerotic cardiovascular disease (ASCVD). The vascular abnormalities associated with impaired glucose metabolism are characterized by an imbalance between vasodilation and vasoconstriction, increased arterial stiffness, activation of atherogenic and pro-thrombotic responses, and abnormal arterial wall remodeling caused by complicated pathophysiological responses such as excess inflammation, activated sympathetic nerves, and proliferation of vascular smooth muscle cells [2, 3]. As a consequence, diabetes accelerates the progression of vascular failure, conferring a doubling in risk for ASCVD, independent from other conventional risk factors [4].

**Fig. 1:**
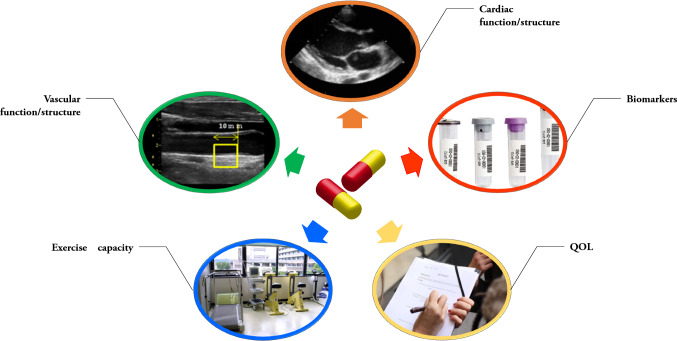
Testing the cardiovascular potentials of drugs through surrogate markers

Whether or not diabetes treatment reduces the incidence of ASCVD has been studied extensively over a long period of time [1, 5–10]. In the last decade, several newer glucose-lowering agents have been tested in large-scale cardiovascular outcome trials (CVOT) to evaluate their cardiovascular safety, prior to commercial distribution [11]. Those clinical trials provided considerable information on the clinical safety and efficacy of each agent, findings that have had a major influence on relevant clinical guidelines and recommendations for daily clinical practice. On the other hand, consistent results have not always been observed within each class of agent and the anticipated cardiovascular benefits estimated from basic research were not always seen in CVOTs. These results indicate the presence of cardiovascular effects in specific patient population studies, but no common or specific effects on cardiovascular function and structure. To better understand these profound cardiovascular effects and further explore the underlying mechanisms which explain the cardiovascular results seen in CVOTs, researchers have examined the findings of clinical trials in greater detail and assessed the impact of agents on various types of cardiovascular surrogate markers (Fig. 1). However, despite using the same class of drugs and similar study designs, the results of the studies are often inconsistent and even conflicting, possibly causing some confusion in daily clinical practice. Based on our previous studies and those of other researchers on the glucose-lowering class of drug, dipeptidyl dipeptidase-4 (DPP-4) inhibitors, this paper discusses the clinical significance and use of cardiovascular surrogate markers to examine the specific cardiovascular effects of cardiometabolic therapies.

## DPP-4 inhibitors and CVOTs

DPP-4 inhibitors are a class of glucose-lowering agents that increase insulin secretion in a blood glucose-dependent manner by increasing the concentration of incretin hormones, such as glucagon-like peptide-1 and glucose-dependent insulinotropic polypeptide. Due to their safe and effective action on glycemic control, DPP-4 inhibitors are now the most frequently prescribed agent in Asian patients with type 2 diabetes (T2D). To date, five CVOTs on DPP-4 inhibitors have reported a comparison of placebo and glimepiride. A pooled meta-analysis of these trials showed a neutral effect on combined major adverse cardiovascular events and their individual components including ASCVD and mortality [12, 13]. Although the enrolled patient population and observation period were different between the five trials, the primary endpoint of assessing non-inferior safety to placebo was met, while the level of safety required by the authorities was achieved. These findings were the main reason for establishing the position of DPP-4 inhibitors in the current market. Meanwhile, real-world data from Korea demonstrated that there may be some differences between DPP-4 inhibitors in terms of the risk of cardiovascular events [14].

## Vascular effects of DPP-4 inhibitors

Due to the design of these CVOTs no information regarding the specific effects on cardiovascular systems was obtained, despite previous studies on DPP-4 inhibitors showing that treatment did not reduce the incidence of ASCVD. However, previous experimental studies demonstrated that DPP-4 inhibitors have anti-atherosclerotic effects [15–18]. To verify these vascular effects in clinical settings, several clinical studies using cardiovascular surrogate markers have been carried out. In this section, we review the clinical studies that have investigated the effect of DPP-4 inhibitors on representative atherosclerotic and vascular functional markers.

Carotid intima-media thickness (IMT) is an independent predictor of ASCVD and acts as a surrogate marker for the degree of progression of systemic arteriosclerosis. Accordingly, it is used widely in clinical settings and is a simple procedure with high reproducibility [19–21]. For example, the measurement is often used in clinical studies to judge therapeutic effects after intervention. We performed a clinical study (PROLOGUE study) to assess the effect of 24 months of sitagliptin treatment on carotid IMT [22]. A total of 463 patients with T2D were randomized equally into sitagliptin or non-DPP-4 inhibitor groups (conventional treatment group), followed by an observation period of 24 months. The primary endpoint of the PROLOGUE study was the change in mean common carotid artery (CCA) IMT 24 months after randomization. Patient backgrounds at the time of study enrollment were matched in both treatment groups, with a mean age of 70 years, HbA1c of approximately 7%, and a history of ASCVD in approximately 50% of the patients. Although sitagliptin tended to suppress the progression of IMT in the PROLOGUE study, there was no significant difference between the two groups in the magnitude of change in mean CCA IMT, despite the safe and efficient hypoglycemic effect of sitagliptin treatment. Other researchers also investigated whether DPP-4 inhibitors attenuated carotid IMT progression in patients with T2D, a background that differed from that of the patients in the PROLOGUE study. The SPEAD-A study showed that 24 months of alogliptin treatment attenuated carotid IMT progression in patients with T2D without a history of ASCVD [23]. The SPIKE study also found that 24 months of sitagliptin treatment attenuated carotid IMT progression in patients with insulin-treated T2D without a history of ASCVD [24].

The results of the primary endpoint were therefore different between our study and these other investigations. We suspect that the different results were due to the different backgrounds of the patients recruited. First, in the PROLOGUE study, HbA1c at the start of treatment was numerically lower than in the other studies, while the target level of HbA1c during the study period was set at 6.2%, allowing aggressive addition of therapeutic agents in both groups. In fact, in the PROLOGUE study some glucose-lowering agents, such as pioglitazone and metformin which suppress the progression of IMT [25, 26], were also administered more frequently in the conventional treatment group. This suggests that addition of some glucose-lowering agents may have masked the effect of sitagliptin on carotid IMT in that study [27]. In addition, the fact that statins had already been introduced at enrollment in subjects in the PROLOGUE study at a frequency of 1.5 to 2 times that used in other studies also contributed to difficulty detecting the effect of sitagliptin. Furthermore, two other studies included patients with no history of ASCVD, whereas about half of the patients in the PROLOGUE trials had a history of ASCVD. We then conducted a subgroup analysis of the primary and secondary prevention groups. We found that sitagliptin treatment partially delayed the progression of carotid IMT in the primary prevention group but did not affect progression in the secondary prevention group [28]. Given these findings, the carotid IMT progression inhibitory effect of DPP-4 inhibitors was more apparent in the primary prevention group, where the progression of arteriosclerosis itself was not considered to be very strong. In contrast, it was speculated that the treatment may have been less effective in the secondary prevention group who possibly had advanced atherosclerosis and even ASCVD. These findings emphasize the importance of early therapeutic intervention before the onset of cardiovascular disease in patients with T2D.

A number of experimental studies have demonstrated that DPP-4 inhibitors improve vascular endothelial function through several molecular and signaling pathways [16, 29–40]. Accordingly, physiological vascular functional tests are also often used to detect vascular failure and monitor therapeutic effects in both daily clinical practice and clinical studies [41]. The PROLOGUE study measured brachial-ankle pulse wave velocity (baPWV) as an index of arterial stiffness and flow-mediated dilation (FMD) as a marker of endothelial function. Overall, there was no significant difference in baPWV levels between the two treatment groups, but interestingly, baPWV decreased over time in patients whose HbA1c was < 7% during the study period, but not in patients whose HbA1c was > 7% [42]. In another substudy of the PROLOGUE study on FMD, 24 months of sitagliptin treatment did not affect endothelial function [43].

Other researchers have also investigated the effect of DPP-4 inhibitors on endothelial function in various clinical settings and study designs (Table 1). Although a detailed review of these studies is outside the scope of this paper, they appear to show inconsistent and even confounding results. Almost all the studies included patients with T2D; however, the detailed medical background of the patients differed between studies to a greater or lesser extent. No study had exactly the same design or target population, suggesting that each result only indicated the potential impact of the research situation for the DPP-4 inhibitor tested and/or the patient population investigated. In addition, whether or not the results can be applied to other research and clinical situations and patient populations is a major limitation and another scientific matter for consideration.

**Table 1:** Comparison of clinical studies that have investigated the effect of DPP-4 inhibitors on endothelial function

DPP-4 inhibitor	Comparator	Observation period	Population	Result	Citation
Sitagliptin	None	12 weeks	T2D	Improved FMD	Kubota Y, et al. J Korean Med Sci. 2012
Sitagliptin	Voglibose	6 weeks	T2D (men)	Decreased FMD	Ayaori M, et al. J Am Heart Assoc. 2013
Alogliptin	(cross-over)	Post-prandial 8 h	Healthy volunteers	Improved FMD	Noda Y, et al. Cardiovasc Diabetol. 2013
Sitagliptin	Conventional therapy	6 months	Uncontrolled T2D and CAD	Improved RHI	Matsubara J, et al. Circ J. 2013
Sitagliptin	Placebo	12 weeks	Newly diagnosed IGT or T2D with ACS	No change in RHI	Hage C, et al. Diab Vasc Dis Res. 2014
Sitagliptin	Voglivose	12 weeks	Uncontrolled T2D	Improved FMD	Nakamura K, et al. Cardiovasc Diabetol. 2014
Teneligliptin	None	3 months	T2D	Improved FMD	Hashikata T, et al. Heart Vessels. 2016
Trelagliptin	None	12 weeks	T2D	No change in FMD	Ida S, et al. Cardiovasc Diabetol. 2016
Sitagliptin	Conventional therapy	24 months	T2D	No change in FMD	Maruhashi T, et al. Cardiovasc Diabetol. 2016
Not specific	Conventional therapy	12 months	T2D	Improved FMD	Leung M, et al. Diab Vasc Dis Res. 2016
Linagliptin	Placebo	12 weeks	T2D	FMD tended to improve	Baltzis D, et al. J Clin Endocrinol Metab. 2016
Linagliptin	Placebo	4 weeks	T2D	Improved renal endothelial function	Ott C, et al. Diabetologia. 2016
Linagliptin	Glimepiride	4 weeks	T2D (no history of ASCVD)	No change in FMD	Jax T, et al. Cardiovasc Diabetol. 2017
Saxagliptin	Metformin	12 weeks	Newly diagnosed T2D	Improved FMD	Li F, et al. Exp Clin Endocrinol Diabetes. 2017
Linagliptin	Metformin	16 weeks	Uncontrolled T2D	Improved FMD	Shigiyama F, et al. J Diabetes Investig. 2017
Vildagliptin	Metformin	12 weeks	T2D	No change in FMD	Kitao N, et al. Cardiovasc Diabetol. 2017
Saxagliptin	Metformin	12 months	T2D	Improved FMD	Dell’Oro R, et al. High Blood Press Cardiovasc Prev. 2017
Linagliptin	Voglibose	12 weeks	Untreated and early stage T2D	Improved RHI	Koyama T, et al. Heart Vessels. 2018
Linagliptin	Placebo	12 weeks	T2D and CAD	No change in FMD	Tripolt NJ, et al. Cardiovasc Diabetol. 2018
Saxagliptin	None	3 months	T2D	Improved FMD	Kajikawa M, et al. Sci Rep. 2019
Vildagliptin	Glibenclamide	12 weeks	T2D and HT without ASCVD	No change in RHI	Cosenso-Martin LN, et al. Diabetes Metab Syndr Obes. 2020

ACS: acute coronary syndrome; ASCVD: atherosclerotic cardiovascular disease; CAD: coronary artery disease; FMD: flow-mediated dilation; HT: hypertension; IGT: impaired glucose tolerance; RHI: reactive hyperemia index; T2D: type 2 diabetes

## Clinical perspectives

**Fig. 2:**
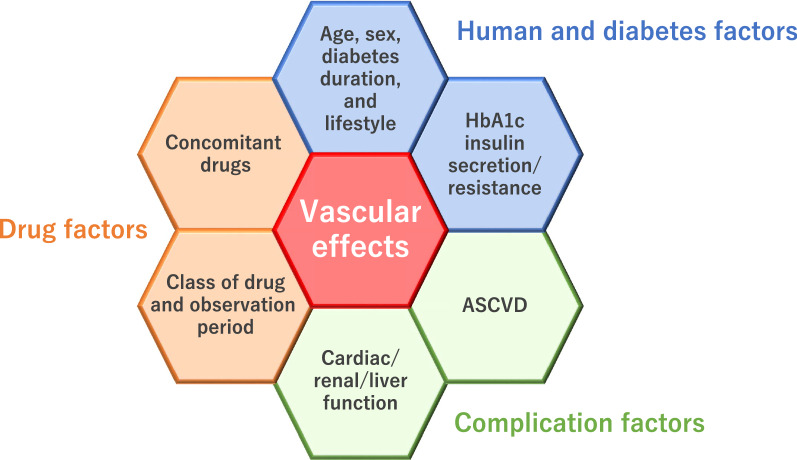
Multifaceted interaction of relevant factors and resultant vascular effect

Clinical studies often use surrogate markers to easily estimate the cardiovascular effects of the drug tested. However, when interpreting the results obtained from such studies it is important to carefully assess the information, taking into account the research situation and the patient population examined. As an example, when assessing the vascular effects of glucose-lowering agents in T2D, it is essential to recognize that the effects observed may often be affected by a number of factors (Fig. 2). If a drug shows a consistent impact on surrogate markers in almost all studies carried out under different situations and/or patient populations, then that impact would be universally expected in a broad range of clinical settings. In contrast, when we are faced with inconsistent or even conflicting impacts on cardiovascular surrogate markers or even differences in the results of CVOTs, it is critical to investigate what caused these differences. This approach can help physicians to predict possible actions and effects of the drug on cardiovascular properties and vascular function in individual patients. Furthermore, because recent clinical studies have reported substantial differences in the risk of atrial fibrillation between classes of glucose-lowering agents [44, 45], further research is also needed to establish potential or suitable surrogate markers of atrial fibrillation and then examine the effect of each drug on these markers. Such approach will result in the optimum selection of drugs and evidence-based patient-centric medication for cardiovascular protection [46].

## Conclusion

The results of large-scale CVOTs and even individual clinical studies have been obtained under very limited conditions for specific endpoints and do not necessarily represent the entire potential of the drug. It is also difficult to make a direct comparison of studies conducted under different conditions and patient populations. Therefore, it is reasonable to consider that a result may show only one aspect of an individual research situation and/or specific patient population. Assessment of the impact on cardiovascular surrogate markers observed in clinical studies from multiple perspectives should help to better understand the potential cardiovascular effects and lead to more appropriate selection of drugs for tailored medication.

## Data Availability

Not applicable.
